# Impact of Left Ventricular Mass Index and Geometric Pattern on Right Ventricular Dimensions and Function in Hypertensive Patients Using Dual-Source Computed Tomography

**DOI:** 10.7759/cureus.109765

**Published:** 2026-05-27

**Authors:** Loay T Soliman, Mohamed Z Elramly, Ahmed Shehatah

**Affiliations:** 1 Cardiology, Cairo University, Cairo, EGY

**Keywords:** computed tomography coronary angiography, hypertension, left ventricular geometry, left ventricular hypertrophy, right ventricular dimensions, right ventricular function

## Abstract

Background

Hypertension is a major and highly prevalent cardiovascular risk factor, affecting approximately 30-45% of the general population, with prevalence increasing markedly with age. It predominantly affects the left ventricle; however, its impact on the right ventricle is less well characterized. Understanding the structural and functional interplay between the two ventricles is essential for comprehensive cardiovascular risk assessment. This study aimed to evaluate the impact of left ventricular mass index (LVMI) and geometric pattern on right ventricular dimensions and systolic function in hypertensive patients using dual-source computed tomography (CT).

Methods

This observational retrospective study included 150 hypertensive patients aged >40 years undergoing CT coronary angiography. LVMI was calculated using semi-automated epicardial and endocardial contouring. Relative wall thickness (RWT) was derived (RWT = 2 × posterior wall thickness/left ventricular internal diameter), with RWT >0.42 considered abnormal for geometric classification. Right ventricular end-diastolic and end-systolic volumes were obtained using semi-automated contour analysis. Right ventricular ejection fraction (RVEF) was calculated from volumetric data, and tricuspid annular plane systolic excursion (TAPSE) was measured using reconstructed images.

Results

The mean LVMI was 74.45 g/m² (±25.47). Overall, 52% had normal geometry, 25% concentric remodelling, 10% eccentric hypertrophy, and 13% concentric hypertrophy. The mean RVEF was 41.58% (±7.39), and the mean TAPSE was 1.67 cm (±0.35). Mean right ventricular end-systolic and end-diastolic volumes were 86.5 mL (±20.87) and 146.5 mL (±30.45), respectively. LVMI demonstrated significant positive correlations with right ventricular volumes and significant negative correlations with RVEF and TAPSE (p < 0.05 for all). Patients with abnormal geometric patterns had significantly larger right ventricular volumes compared with normal geometry (p < 0.05). Right ventricular systolic function progressively declined from normal geometry to concentric hypertrophy. Post-hoc analysis showed significant differences between most geometric groups, except between eccentric and concentric hypertrophy.

Conclusions

Left ventricular hypertrophy and abnormal geometric patterns in hypertension are associated with progressive right ventricular dilatation and systolic impairment, suggesting early bi-ventricular involvement in hypertensive heart disease. Early identification of left ventricular remodelling may be crucial to prevent secondary right ventricular dysfunction, a well-established predictor of adverse cardiovascular outcomes.

## Introduction

Hypertension is a major and highly prevalent cardiovascular risk factor, affecting approximately 30-45% of the general population, with prevalence increasing markedly with age [1]. The effects of hypertension on the left ventricle have been well established; however, the structural and functional consequences on the right ventricle have received comparatively limited attention, partly due to its complex geometry and orientation [2]. Although previous echocardiographic studies have evaluated aspects of right ventricular (RV) involvement in hypertension, data using computed tomography (CT)-derived volumetric and functional assessment in relation to left ventricular (LV) geometric patterns remain limited. LV hypertrophy, defined as an increase in LV mass, is a common manifestation of hypertension and is associated with adverse cardiovascular outcomes [3]. In addition, distinct LV geometric patterns have been identified as important prognostic markers in hypertensive patients. Despite this, the impact of left ventricular mass index (LVMI) and LV geometric patterns on RV dimensions and systolic function in hypertensive patients remains incompletely understood.

RV function is a major determinant of prognosis in many cardiovascular disorders [4]. However, its assessment is technically challenging due to the complex shape of the RV and its thin free wall. Echocardiography is commonly used for RV evaluation but has limitations, including operator dependency and suboptimal image quality in certain patients. Although cardiac magnetic resonance imaging (MRI) is considered the reference standard for RV assessment, it is not universally available, may be costly, and is unsuitable for some patients, particularly those with claustrophobia or metallic implants. Dual-source CT is a non-invasive imaging modality with accuracy comparable to cardiac MRI and provides superior spatial resolution compared with echocardiography, allowing improved endocardial border delineation [5,6]. Although radiation exposure and iodinated contrast administration are potential disadvantages, recent advances in dose-reduction strategies and contrast optimization techniques have substantially mitigated these concerns.

The association between arterial hypertension and RV remodelling may be explained by ventricular interdependence, particularly through the interventricular septum, which plays a central role in RV systolic performance [7]. Potential mechanisms proposed in previous studies include retrograde transmission of elevated LV diastolic pressure, increased pulmonary vascular resistance, and neurohormonal activation. Overstimulation of the renin-angiotensin-aldosterone system (RAAS), sympathetic nervous system activation, endothelial dysfunction, oxidative stress, and increased production of growth factors may further contribute to RV remodelling in hypertensive patients. Previous studies have demonstrated higher plasma renin activity and aldosterone levels in hypertensive patients with eccentric and concentric LV hypertrophy [8,9]. Furthermore, improvement in RV function following antihypertensive therapy with renin-angiotensin system inhibitors supports the hypothesis that RAAS plays an important role in RV remodelling [8].

Given the established prognostic importance of RV function and the recognized impact of LV remodelling in hypertension, clarifying the relationship between LVMI, LV geometry, and RV structure and function is clinically relevant. Therefore, this study aimed to investigate the association between LVMI and RV dimensions and systolic function, and to evaluate the relationship between LV geometric patterns and RV remodelling in hypertensive patients using dual-source CT.

## Materials and methods

Study design and population

This retrospective, cross-sectional, observational study enrolled 150 patients with primary arterial hypertension who were referred for CT coronary angiography to exclude coronary artery disease. Patients were consecutively recruited from individuals referred to a single tertiary care centre during the study period, from January 2025 to December 2025. The study protocol was approved by the local institutional ethics committee, and all participants provided written informed consent for use of anonymized clinical and imaging data for research purposes before inclusion in the study.

Patients aged >40 years with a prior diagnosis of primary hypertension who were referred for CT coronary angiography were eligible for inclusion. Hypertension was defined as a prior clinical diagnosis of primary hypertension, current antihypertensive treatment, and/or office blood pressure ≥140/90 mmHg based on standard diagnostic criteria at the time of clinical assessment. Baseline blood pressure measurements and antihypertensive treatment status were recorded for all patients. Exclusion criteria included chronic obstructive pulmonary disease, previously established coronary artery disease (including prior myocardial infarction, percutaneous coronary intervention, or coronary artery bypass grafting), chronic kidney disease (eGFR: <60 mL/minute/1.73 m²), known allergy to iodinated contrast media, more than moderate valvular heart disease, and refusal to participate in the study.

All patients underwent baseline electrocardiography (ECG) and transthoracic echocardiography before inclusion. Patients were excluded if echocardiography demonstrated significant pulmonary hypertension, defined as estimated pulmonary artery systolic pressure >40 mmHg or intermediate/high echocardiographic probability of pulmonary hypertension, reduced LV systolic function (left ventricular ejection fraction (LVEF): <50%), congenital heart disease, or features suggestive of cardiomyopathy. Patients with atrial fibrillation or other significant arrhythmias on baseline ECG were also excluded.

Clinical assessment included detailed medical history and physical examination, with documentation of cardiovascular risk factors, including diabetes mellitus, smoking status, and duration of hypertension. Body surface area (BSA) was calculated using the Mosteller formula.

Computed tomography acquisition protocol

All CT examinations were performed using the 384 multi-detector row scanner (Siemens SOMATOM force, Erlangen, Germany). Scanning parameters were: 192 × 0.25 mm collimation, tube rotation time of 250 ms, tube voltage of 70 kV (increased up to 120 kV in obese patients), and current of 292-600 mA. The field of view was 35.4 cm with an image matrix of 512 × 512 pixels up to 1,024 × 1,024 pixels. The scanning direction was craniocaudal.

For contrast enhancement, a weight-based volume of non-ionic contrast medium at an average of 75 mL (Ultravist 370 Schering, Berlin, Germany) was injected through an 18-gauge cannula into an upper limb vein (right antecubital vein is preferred when available) with a flow rate of 5-6 mL/second using a programmed dual-head injector pump (MedRad; USA). This was followed by an injection of 40 mL of chasing saline to push the injected contrast material and to wash out the right side of the heart.

The patient was placed in a supine position on the CT table. ECG leads were fixed at their positions on the chest wall. Images were reconstructed throughout the cardiac cycle using retrospective ECG gating. End-diastolic phases were typically selected at approximately 70-80% of the R-R interval, while end-systolic phases were selected at approximately 30-40%, according to the phases demonstrating maximal and minimal ventricular cavity dimensions, respectively. First, a localization scan (topogram) was performed that yielded anteroposterior (AP) and lateral views of the chest and was used to position the imaging volume of the coronary arteries that extended from the level of the carina down to about 1 cm below the diaphragm. The center of the field of view was 2 cm to the left of the dorsal spine on the AP scout and at the level of the hilum on the lateral scout. To minimize the total effective patient radiation dose, this stage of the scanning was conducted with a relatively low tube current. Semi- automated determination of the starting time using the bolus-tracing technique was utilized in all patients. Ventricular analysis was performed using commercially available software (syngo.via, Siemens Healthineers, Erlangen, Germany).

Left ventricular assessment

**Figure 1:**
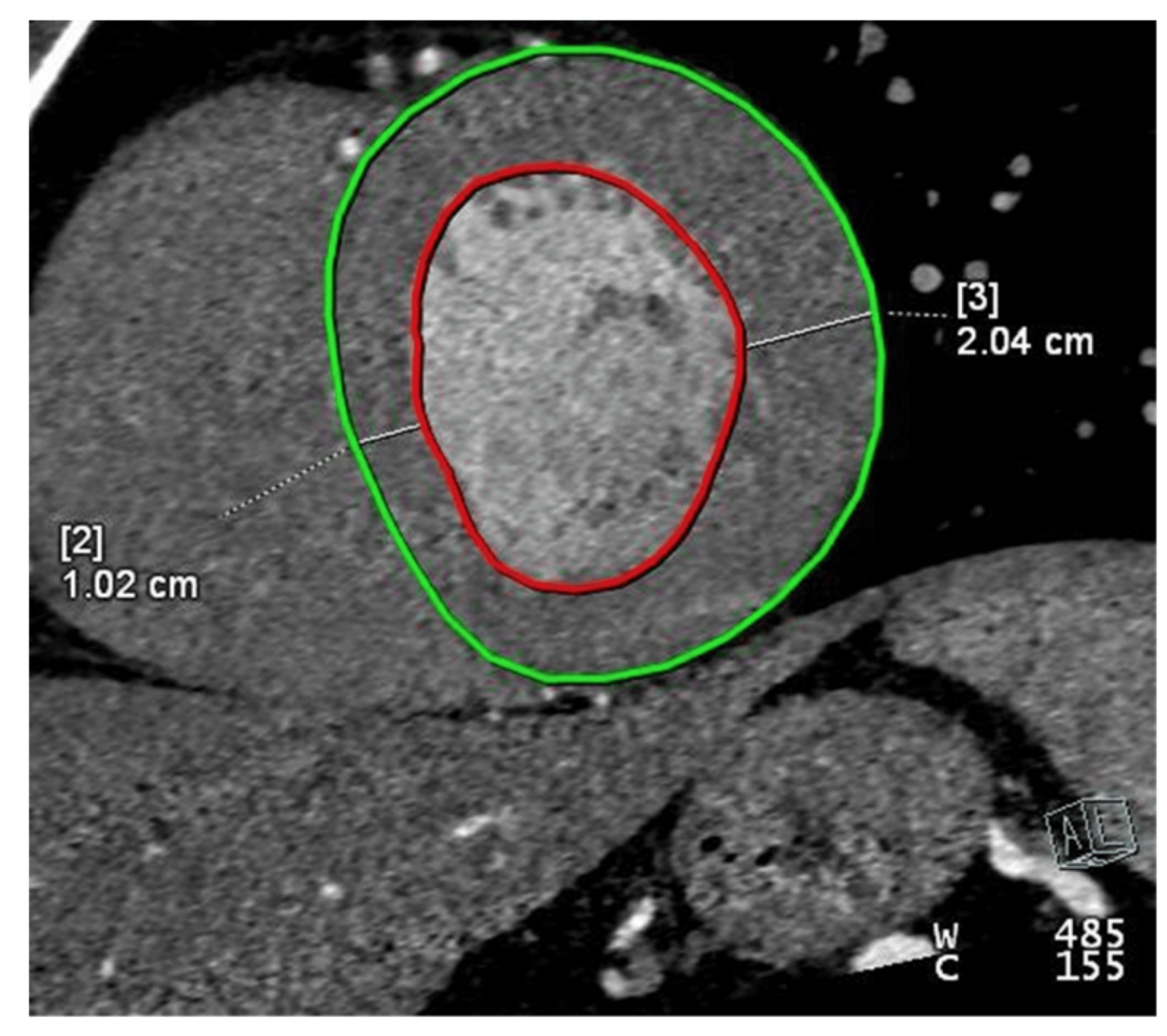
Left ventricular assessment. Short-axis view of the left ventricle with endocardial border in red and epicardial border in green used to calculate left ventricular mass, and the septal and posterior wall thickness.

As depicted in Figure 1, multiplanar reformatted images (short-axis, two-chamber, and four-chamber views) were used for LV analysis. LV endocardial and epicardial borders were automatically detected using dedicated software with manual correction when necessary to exclude papillary muscles.

LV mass was calculated from the difference between epicardial and endocardial volumes and multiplied by myocardial density (1.04 g/mL). LV mass was indexed to BSA to obtain the LVMI. LV hypertrophy was defined as greater than 89 g/m² in women and greater than 103 g/m² in men [10,11].

Posterior wall thickness and LV internal diameter were measured at end-diastole. LV internal diameter was measured at the level of the chordae on the four-chamber multi-planar reformatted image, while posterior wall thickness was measured on a short-axis view. Relative wall thickness (RWT) was calculated as RWT = 2 × posterior wall thickness/LV internal diameter. An RWT >0.42 was considered abnormal.

As illustrated in Table 1, patients were categorized into four LV geometric patterns: normal geometry (normal LVMI, normal RWT), concentric remodelling (normal LVMI, elevated RWT), eccentric hypertrophy (elevated LVMI, normal RWT), and concentric hypertrophy (elevated LVMI, elevated RWT).

**Table 1:** Classification of left ventricular geometric patterns according to LVMI and RWT. LV = left ventricular; LVMI = left ventricular mass index; RWT = relative wall thickness

LV geometric pattern	LVMI	RWT
Normal geometry	Normal	Normal
Concentric remodelling	Normal	Increased
Eccentric hypertrophy	Increased	Normal
Concentric hypertrophy	Increased	Increased

Right ventricular assessment

**Figure 2:**
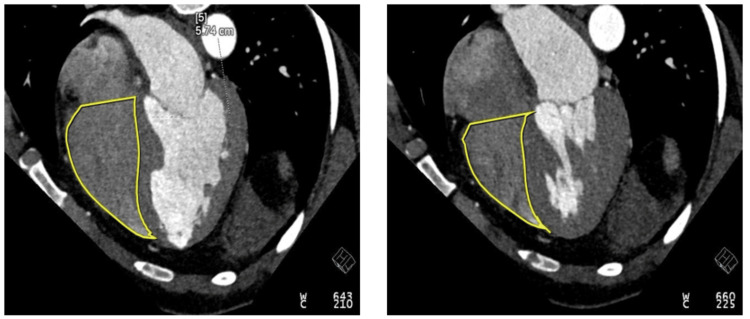
Right ventricular volume assessment. Four-chamber view in systole and diastole with the yellow line along the endocardial border used to measure right ventricular volumes and calculate right ventricular ejection fraction.

As depicted in Figure 2, RV volumes were obtained using multiplanar reformatted images. RV segmentation was performed from the apex to the level of the main pulmonary artery bifurcation. Endocardial contours were generated semi-automatically with manual correction where required, and trabeculations and papillary muscles were excluded from volumetric measurements.

Measured parameters included right ventricular end-diastolic volume (RVEDV), right ventricular end-systolic volume (RVESV), stroke volume (end-diastolic volume − end-systolic volume), and right ventricular ejection fraction (RVEF).

**Figure 3:**
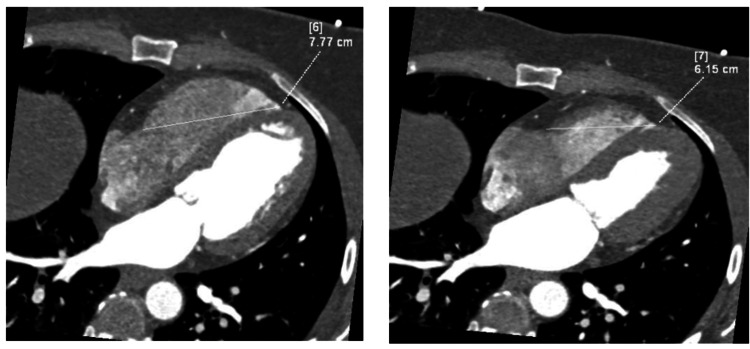
TAPSE measurement. Four-chamber view in systole and diastole depicting TAPSE measurement by measuring the tricuspid annular displacement in relation to the apex. TAPSE = tricuspid annular plane systolic excursion

As seen in Figure 3, RV systolic function was additionally assessed by tricuspid annular plane systolic excursion (TAPSE) measured from four-chamber views by measuring the displacement of the tricuspid annulus in relation to the RV apex in systole and diastole. CT-derived TAPSE has previously been shown to correlate with both CT-derived and MRI-derived RVEF and was therefore used as an additional parameter of longitudinal RV systolic function [12,13]. A TAPSE value <1.7 cm was considered indicative of impaired RV systolic function according to established echocardiographic reference criteria.

Statistical analysis

Statistical analysis was performed using SPSS Statistics version 27 (IBM Corp., Armonk, NY, USA). Continuous variables were expressed as mean ± standard deviation, while categorical variables were presented as frequencies and percentages. Comparisons between LV geometric groups were performed using one-way analysis of variance (ANOVA) with least significant difference (LSD) post-hoc testing for exploratory subgroup comparisons. Associations between continuous variables were evaluated using Pearson correlation analysis, and categorical variables were compared using the chi-square test. A p-value <0.05 was considered statistically significant.

Reproducibility analysis

To assess measurement reproducibility, CT data from the first 20 patients were reanalyzed one week later to evaluate intraobserver variability and by a second observer to assess interobserver variability. Correlation coefficients ranged from 0.88 to 0.98 for intraobserver and 0.89 to 0.97 for interobserver measurements, indicating high reproducibility.

## Results

Baseline characteristics

A total of 150 hypertensive patients were included in the study. As depicted in Table 2, the mean age of the cohort was 59.3 ± 9.8 years, and 90 (60%) patients were male. Overall, 56 (37.3%) patients were smokers, and 51 (34%) patients had diabetes mellitus. The mean systolic blood pressure was 139 ± 13 mmHg, while the mean diastolic blood pressure was 78 ± 9 mmHg. The average duration of hypertension was 9.2 ± 6.5 years. Regarding antihypertensive therapy, 72 (48%) patients were receiving monotherapy, while 72 (48%) patients were on combination therapy (dual, triple, or quadruple therapy).

**Table 2:** Demographic and clinical characteristics of the study group. Values are presented as mean ± standard deviation or n (%).

Variable	Value
Age, years	59.34 ± 9.78
Weight, kg	91.27 ± 18.00
Height, cm	166.98 ± 7.58
Body surface area, m²	2.05 ± 0.22
Duration of hypertension, years	9.21 ± 6.47
Systolic blood pressure, mmHg	139.07 ± 13.25
Diastolic blood pressure, mmHg	78.10 ± 9.34
Male sex	90 (60.0%)
Female sex	60 (40.0%)
Smokers	56 (37.3%)
Diabetes mellitus	51 (34.0%)
Antihypertensive medications (n)	
0	6 (4.0%)
1	72 (48.0%)
2	48 (32.0%)
3	21 (14.0%)
4	3 (2.0%)

Left ventricular computed tomography findings

Table 3 illustrates the CT data of LV analysis. Based on LVMI and RWT, patients were classified into four LV geometric patterns: 77 (51.3%) patients had normal geometry, 38 (25.3%) patients had concentric remodelling, 15 (10%) patients had eccentric hypertrophy, and 20 (13.3%) patients had concentric hypertrophy. Patients with abnormal LV geometric patterns were significantly older, had a longer duration of hypertension, and demonstrated higher systolic blood pressure compared with patients with normal geometry (p < 0.001).

**Table 3:** CT data of LV analysis. CT = computed tomography; LV = left ventricular; LVM = left ventricular mass; LVMI = left ventricular mass index; RWT = relative wall thickness

	Mean	SD
LVM (g)	152.25	53.48
LVMI (g/m²)	74.45	25.37
RWT	0.41	0.062

Table 4 illustrates the different clinical characteristics and RV measurements across the four LV geometric sub-groups.

**Table 4:** Clinical and demographic characteristics within each LV geometric pattern. BSA = body surface area; HTN = hypertension; DM = diabetes mellitus; SBP = systolic blood pressure; DBP = diastolic blood pressure; LV = left ventricular; LVMI = left ventricular mass index; RWT = relative wall thickness; RV = right ventricular; RVEDV = right ventricular end-diastolic volume; RVESV = right ventricular end-systolic volume; RVEF = right ventricular ejection fraction; TAPSE = tricuspid annular plane systolic excursion

Variable	Normal (n = 77)	Concentric remodelling (n = 38)	Eccentric hypertrophy (n = 15)	Concentric hypertrophy (n = 20)	P-value
Clinical characteristics					
Age (years)	55.64 ± 8.40	58.89 ± 8.93	67.93 ± 7.59	68.00 ± 8.73	<0.001
Gender (Male/Female)	43/34	24/14	10/5	13/7	0.753
BSA (m²)	2.03 ± 0.21	2.10 ± 0.24	2.02 ± 0.19	2.02 ± 0.19	0.545
Duration of HTN (years)	5.57 ± 2.43	9.68 ± 5.34	15.53 ± 6.15	17.55 ± 7.87	<0.001
DM, n (%)	21	17	3	10	0.069
Smokers, n (%)	33	12	3	8	0.317
Number of medications					0.008
0	1	3	0	2	
1	46	14	6	6	
2	25	15	4	4	
3	5	5	4	7	
4	0	1	1	1	
Hemodynamic parameters					
SBP (mmHg)	134.94 ± 10.50	139.87 ± 12.00	146.00 ± 14.17	148.25 ± 17.64	<0.001
DBP (mmHg)	77.01 ± 8.75	78.68 ± 9.91	84.33 ± 9.04	76.50 ± 9.33	0.036
LV structural parameters					
LVMI (g/m²)	61.27 ± 12.53	64.39 ± 11.98	111.33 ± 8.17	116.65 ± 15.43	<0.001
RWT	0.37 ± 0.03	0.47 ± 0.05	0.39 ± 0.02	0.48 ± 0.05	<0.001
RV structural and functional parameters					
RVEDV (mL)	136.61 ± 26.29	149.99 ± 28.19	164.60 ± 29.71	164.45 ± 35.95	<0.001
RVESV (mL)	72.61 ± 16.23	92.29 ± 19.38	111.27 ± 21.01	110.80 ± 28.09	<0.001
RVEF (%)	47.02 ± 4.96	38.70 ± 3.57	32.47 ± 3.50	33.00 ± 4.00	<0.001
TAPSE (cm)	1.90 ± 0.27	1.54 ± 0.25	1.33 ± 0.27	1.30 ± 0.17	<0.001

Right ventricular computed tomography findings

As illustrated in Table 5, the mean RVEDV was 146.5 ± 30.5 mL, while the mean RVESV was 86.6 ± 24.9 mL. The mean RVEF was 41.6 ± 7.4%, and the average TAPSE was 1.67 ± 0.36 cm.

**Table 5:** CT data of RV analysis. CT = computed tomography; RV = right ventricular; RVEDV = right ventricular end-diastolic volume; RVESV = right ventricular end-systolic volume; RVEF = right ventricular ejection fraction; TAPSE = tricuspid annular plane systolic excursion

	Mean	SD
RVEDV (mL)	146.51	30.45
RVESV (mL)	86.55	24.88
RVEF (%)	41.59	7.39
TAPSE (cm)	1.67	0.36

Using an RVEF cutoff of 42% to define RV impairment [14], 75 (50%) patients were classified as having impaired RV function. Patients with RV impairment were significantly older, had a longer duration of hypertension, and demonstrated higher systolic blood pressure and LVMI compared with patients with preserved RV function (p < 0.001).

Table 6 illustrates the demographic and clinical characteristics of participants with and without RV impairment.

**Table 6:** Comparison between patients based of RV impairment. BSA = body surface area; HTN = hypertension; DM = diabetes mellitus; SBP = systolic blood pressure; DBP = diastolic blood pressure; LVMI = left ventricular mass index; LVID = left ventricular internal diameter; RWT = relative wall thickness; RV = right ventricular

		RV not impaired (n = 75)	RV impaired (n = 75)	P-value
Age (years)		55.25 (±8.50)	63.43 (±9.30)	<0.001
Gender	Male	44	46	0.868
	Female	31	29	
BSA (m^2^)		2.04 (±0.22)	2.06 (±0.22)	0.445
Duration of HTN (years)		5.64 (±2.75)	12.77 (±7.13)	<0.001
Number of HTN medications	0	1	5	0.001
	1	46	26	
	2	24	24	
	3	4	17	
	4	0	3	
DM		20	31	0.058
Smokers		32	24	0.177
SBP (mmHg)		135 (±9.83)	143.13 (±14.95)	<0.001
DBP (mmHg)		76.40 (±9.60)	79.80 (±8.80)	0.025
LVMI (g/m^2^)		61.17 (±12.70)	87.73 (±27.90)	<0.001
LVID (cm)		4.46 (±0.35)	4.58 (±0.43)	0.06
RWT		0.38 (±0.05)	0.45 (±0.06)	<0.001
Normal geometry		69	8	<0.001
Concentric remodelling		6	32	<0.001
Eccentric hypertrophy		0	15	<0.001
Concentric hypertrophy		0	20	<0.001

Correlation between left ventricular mass index and right ventricular parameters

**Figure 4:**
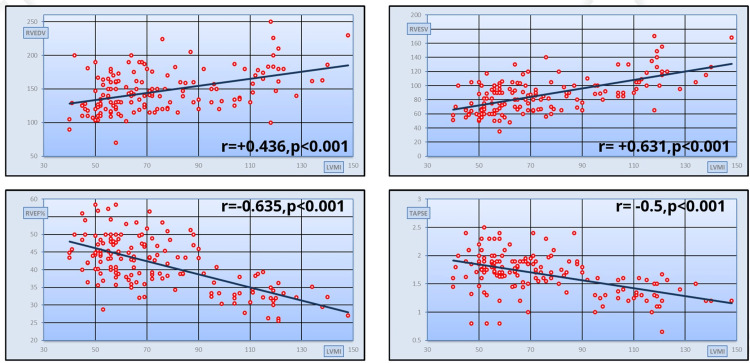
Correlation analysis between LVMI and RVEDV, RVESV, RVEF, and TAPSE. LVMI correlated negatively with RVEF (r = –0.635) and TAPSE (r = –0.500), and positively with RVESV (r = +0.613) and RVEDV (r = +0.436). LVMI = left ventricular mass index; RVEDV = right ventricular end-diastolic volume; RVESV = right ventricular end-systolic volume; RVEF = right ventricular ejection fraction; TAPSE = tricuspid annular plane systolic excursion

As depicted in Figure 4, correlation analysis demonstrated a significant negative correlation between LVMI and RV systolic function, including RVEF (r = −0.635, p < 0.001) and TAPSE (r = −0.500, p < 0.001). LVMI also showed positive correlations with RV volumes, including RVESV (r = 0.613, p < 0.001) and RVEDV (r = 0.436, p < 0.001). These findings indicate that increasing LV mass is associated with greater RV dilation and systolic dysfunction.

Association between left ventricular geometry and right ventricular function

**Figure 5:**
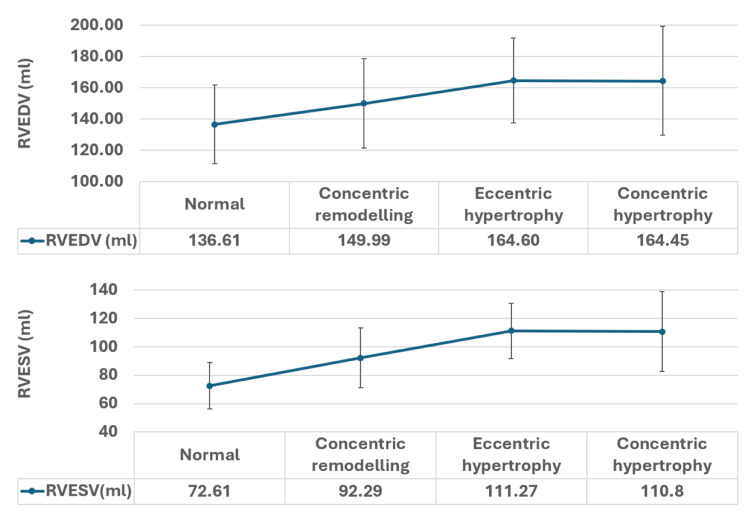
Mean plots of analyses of variance for RVESV and RVEDV. RVEDV differed significantly across subgroups (p < 0.001), driven mainly by differences between normal geometry and eccentric hypertrophy (p = 0.001), concentric remodelling (p = 0.019), and concentric hypertrophy (p < 0.001). RVESV also differed significantly (p < 0.001), mainly between normal vs. abnormal patterns (all p < 0.001), and between concentric remodeling vs. eccentric hypertrophy (p = 0.002) or concentric hypertrophy (p = 0.001). Error bars represent standard deviation (SD). RVEDV = right ventricular end-diastolic volume; RVESV = right ventricular end-systolic volume

As seen in Figure 5, there was a statistically significant difference in RVEDV across the four LV geometric patterns (p < 0.001). Post-hoc analysis demonstrated that the significance was primarily driven by differences between patients with normal LV geometry and those with abnormal geometric patterns, while comparisons between the abnormal geometric patterns were not statistically significant. There was also a statistically significant difference in RVESV across the four LV geometric patterns (p < 0.001). Post-hoc analysis showed significant differences between normal geometry and all abnormal patterns, as well as between concentric remodelling and both eccentric hypertrophy and concentric hypertrophy.

**Figure 6:**
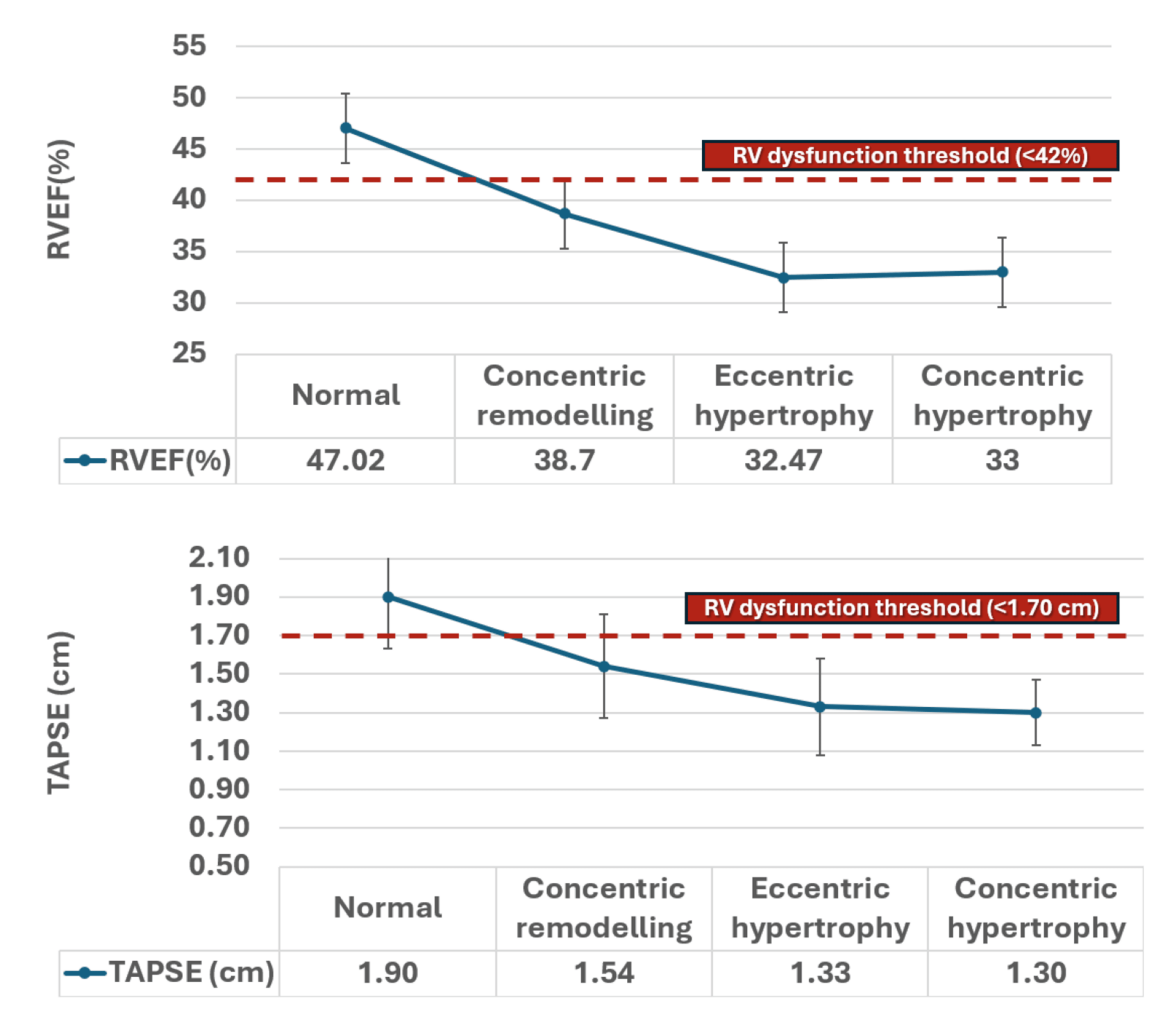
Mean plots of analysis of variance for RVEF and TAPSE. RVEF: Significant difference across subgroups (p < 0.001), driven by normal vs. abnormal patterns, and concentric remodelling vs. eccentric or concentric hypertrophy. Other comparisons not significant. Error bars represent standard deviation (SD). TAPSE: Significant difference across subgroups (p < 0.001), driven by normal vs. abnormal patterns, and concentric remodeling vs. eccentric or concentric hypertrophy. Other comparisons not significant. Error bars represent standard deviation (SD). RVEF = right ventricular ejection fraction; TAPSE = tricuspid annular plane systolic excursion

As depicted in Figure 6, both RVEF and TAPSE demonstrated statistically significant differences across the four LV geometric patterns (p < 0.001). Post-hoc analysis revealed that both parameters were significantly lower in all abnormal LV geometric patterns compared with normal geometry, with additional significant differences between concentric remodelling and both eccentric and concentric hypertrophy.

Predictors of computed tomography-derived right ventricular functional parameters

Multivariable linear regression analysis was performed using age, systolic blood pressure, diastolic blood pressure, LVMI, posterior wall thickness, septal wall thickness, RWT, left ventricular internal diameter, diabetes mellitus, duration of hypertension, number of antihypertensive medications, and LV geometric pattern as covariates. The LV geometric pattern was entered into the regression model as a categorical variable representing the four predefined geometric groups. The overall model was statistically significant (R = 0.744, R² = 0.554, p < 0.001). LV geometric pattern (β = 0.625, p < 0.001) and RWT (β = 0.182, p = 0.007) emerged as the strongest independent predictors of RV impairment.

## Discussion

Arterial hypertension is a highly prevalent cardiovascular risk factor affecting approximately 30-45% of the adult population worldwide, with prevalence increasing significantly with age. While the structural and functional consequences of hypertension on the left ventricle have been extensively studied, the impact of hypertension on the right ventricle has received comparatively less attention (2). The present study investigated the relationship between LVMI and LV geometric patterns with RV dimensions and systolic function using dual-source CT.

To our knowledge, this is one of the few studies to investigate the relationship between LVMI and LV geometric patterns with RV structure and function using dual-source CT. Dual-source CT offers several advantages for cardiac structural assessment compared with transthoracic echocardiography, including reduced operator dependency, superior spatial resolution, and improved delineation of endocardial borders. Although cardiac MRI remains the gold standard for the assessment of RV volumes and function, its use may be limited in patients with implantable devices, claustrophobia, or limited availability in routine clinical practice. Previous studies have demonstrated good agreement between CT- and cardiac MRI-derived measurements of ventricular volumes and function [5,6]. While CT involves exposure to ionizing radiation and iodinated contrast, recent advances in dose-reduction techniques and optimized contrast protocols have substantially mitigated these concerns [15-17].

The principal findings of this study were that LVMI was significantly associated with RV structural and functional parameters (increasing LVMI correlated negatively with RV systolic function, reflected by reductions in RVEF and TAPSE, and positively with RV volumes), abnormal LV geometric patterns were associated with significant alterations in RV volumes and systolic function, and LV geometric pattern and RWT emerged as independent predictors of RV impairment. The relatively high prevalence of RV impairment observed in the present cohort may reflect the selected hypertensive population as well as differences in CT-derived RV functional assessment and threshold definitions across imaging modalities.

Our results demonstrated a significant negative correlation between LVMI and RV systolic function, as measured by RVEF and TAPSE, alongside positive correlations with RVESV and RVEDV. These findings suggest that increasing LV mass is associated with progressive RV dilation and systolic dysfunction. Similar observations have been reported in previous studies. Hamdy et al. demonstrated significant negative correlations between LVMI and RV systolic function parameters, including RV fractional area change, TAPSE, and RVEF, using four-dimensional echocardiography [4]. Likewise, Ojji et al. reported a negative association between LVMI and TAPSE in patients with hypertensive heart failure [18]. Li et al. also observed that patients with increased LVMI exhibited significantly lower RV systolic function parameters compared with hypertensive patients with normal LVMI [19]. Collectively, these findings support the concept that LV hypertrophy in hypertension may be associated with subclinical RV dysfunction.

In addition to LV mass, our study demonstrated that LV geometric pattern significantly influenced RV structure and function. Patients with abnormal LV geometry exhibited significantly higher RV volumes and reduced RV systolic function compared with those with normal geometry. Post-hoc analysis indicated that these differences were primarily driven by comparisons between normal geometry and abnormal geometric patterns. Similar findings have been reported by Tadic et al. and Xue et al., who demonstrated step-wise associations between LV geometric remodelling patterns and RV systolic dysfunction in patients with concentric and eccentric LV hypertrophy [7,20]. These studies suggest that advanced LV remodelling is associated with structural and functional alterations in the right ventricle.

Multivariable regression analysis in our study identified LV geometric pattern and RWT as independent predictors of RV impairment. These findings are consistent with those reported by Tadic et al., who also identified abnormal LV geometry as a key determinant of RV dysfunction in hypertensive patients [7]. In contrast, Xue et al. reported that LVMI, systolic blood pressure, and RWT were independent predictors of RV dysfunction [20]. Differences in methodology and the specific RV functional parameters assessed may partially explain these discrepancies.

Several pathophysiological mechanisms may explain the association between LV remodelling and RV dysfunction in hypertension. Ventricular interdependence plays a central role in this relationship, as the interventricular septum contributes significantly to RV systolic performance [7]. Structural and functional changes in the left ventricle may therefore influence RV mechanics through septal interaction. In addition, chronic hypertension is associated with activation of neurohormonal pathways, including the RAAS and the sympathetic nervous system, which may promote myocardial hypertrophy, fibrosis, and adverse ventricular remodelling [9]. Elevated pulmonary pressures secondary to increased LV filling pressures may also contribute to progressive RV structural changes in hypertensive patients [9].

This study has several limitations that should be considered. The sample size was relatively modest, with a limited number of patients within some LV geometric pattern subgroups, which may affect statistical power. In addition, the study was conducted at a single center, potentially limiting the generalizability of the findings. Although CT provides excellent spatial resolution and reproducible volumetric measurements, RV function was not validated against a reference imaging modality such as cardiac MRI, despite previously reported agreement between cardiac CT and cardiac MRI-derived ventricular measurements in the literature discussed before. Multiple statistical comparisons were performed, which may increase the risk of type I error, particularly for secondary subgroup analyses. In addition, the relatively high prevalence of RV impairment observed in the present cohort may have been influenced by referral bias related to the inclusion of patients undergoing CT coronary angiography, CT-derived functional assessment methodology, RV functional cutoff selection, and residual confounding from clinical variables such as age, hypertension duration, systolic blood pressure, diabetes mellitus, and medication burden. However, the principal associations between LV geometric remodelling parameters and RV structural and functional measures demonstrated strong statistical significance, with most primary findings showing p-values <0.001. Although reproducibility analysis demonstrated high measurement consistency, intraclass correlation coefficients and Bland-Altman analysis were not performed, which may have provided a more comprehensive assessment of inter and intraobserver agreement. Confidence intervals for correlation coefficients were not reported, which may limit assessment of the precision of the observed associations. Finally, the cross-sectional design of the study precludes establishing causal relationships between LV remodelling and RV dysfunction.

## Conclusions

This study highlights the association between LV structural remodelling and RV structure and function in patients with hypertension. The findings suggest that hypertensive cardiac remodelling may involve both ventricles rather than being limited to the left ventricle alone. These observations emphasize the association between hypertensive cardiac remodelling and RV structural and functional changes, highlighting the potential clinical relevance of early recognition and optimal hypertension management.
